# Medial Collateral Ligament Deficiency of the Elbow Joint: A Computational Approach

**DOI:** 10.3390/bioengineering5040084

**Published:** 2018-10-10

**Authors:** Munsur Rahman, Akin Cil, Antonis P. Stylianou

**Affiliations:** 1 Department of Civil and Mechanical Engineering, University of Missouri-Kansas City, 5110 Rockhill Road, Kansas City, MO 64110, USA; Akin.Cil@tmcmed.org (A.C.); stylianoua@umkc.edu (A.P.S.); 2 Department of Orthopaedic Surgery, University of Missouri-Kansas City, 2411 Holmes Street, Kansas City, MO 64108, USA; 3 Department of Orthopaedics, Truman Medical Centers, 2301 Holmes Street, Kansas City, MO 64108, USA

**Keywords:** elbow joint, medial collateral ligament, ligament deficiency, computational model, kinematics, upper extremity

## Abstract

Computational elbow joint models, capable of simulating medial collateral ligament deficiency, can be extremely valuable tools for surgical planning and refinement of therapeutic strategies. The objective of this study was to investigate the effects of varying levels of medial collateral ligament deficiency on elbow joint stability using subject-specific computational models. Two elbow joint models were placed at the pronated forearm position and passively flexed by applying a vertical downward motion on humeral head. The models included three-dimensional bone geometries, multiple ligament bundles wrapped around the joint, and the discretized cartilage representation. Four different ligament conditions were simulated: All intact ligaments, isolated medial collateral ligament (MCL) anterior bundle deficiency, isolated MCL posterior bundle deficiency, and complete MCL deficiency. Minimal kinematic differences were observed for isolated anterior and posterior bundle deficient elbows. However, sectioning the entire MCL resulted in significant kinematic differences and induced substantial elbow instability. Joint contact areas were nearly similar for the intact and isolated posterior bundle deficiency. Minor differences were observed for the isolated anterior bundle deficiency, and major differences were observed for the entire MCL deficiency. Complete elbow dislocations were not observed for any ligament deficiency level. As expected, during isolated anterior bundle deficiency, the remaining posterior bundle experiences higher load and vice versa. Overall, the results indicate that either MCL anterior or posterior bundle can provide anterior elbow stability, but the anterior bundle has a somewhat bigger influence on joint kinematics and contact characteristics than posterior one. A study with a larger sample size could help to strengthen the conclusion and statistical significant.

## 1. Introduction

Elbow dislocations are very common in adults and children and represent 11–28% of all elbow injuries at an average annual rate of 6 to 13 cases per 100,000 population [1,2]. Dislocations of the elbow can be simple, where the dislocation occurs with the medial collateral ligament (MCL) and/or lateral collateral ligament injury or can be complex, where the dislocation causes both ligament injury and fracture of the articular surface [2].

The MCL provides resistance to valgus motion of the elbow joint. While the bony articulation contributes to stability, the MCL complex provides the primary medial elbow stabilization. In acute elbow dislocations, MCL injury has been reported as high as 100% [3]. Occupations or activities, where the elbow is placed under repetitive valgus load, can produce MCL microtears and eventually complete disruption of the MCL [4]. MCL injury can produce chronic pain in the medial aspect of the elbow and can result in problems in other surrounding areas such as the ulnar nerve, the flexor-pronator musculotendinous unit, the radiocapitellar joint, and the posterior compartment of the elbow [5]. MCL deficiency can cause significant joint morbidity and may end the career for overhead athletes such as baseball pitchers, and javelin throwers.

Simulating medial collateral ligament deficiency in a computer model would be immensely valuable for surgical planning and refinement of rehabilitation regimens. Such models would allow us to examine efficient ligament reconstruction techniques by pre-operative assessment and to investigate better rehabilitation post-operative protocols. Literature reviews reveal that computer models have been employed effectively to measure articular cartilage contact pressure distribution, examine muscle and ligament function, investigate joint stability, and injury mechanisms [6,7,8,9,10,11,12,13,14,15,16,17,18]. Computer models provide great flexibility in analyzing different clinical scenarios and are capable of measuring and calculating important parameters that are difficult or sometimes impossible to capture experimentally such as ligament force and cartilage contact pressure.

**Figure 1:**
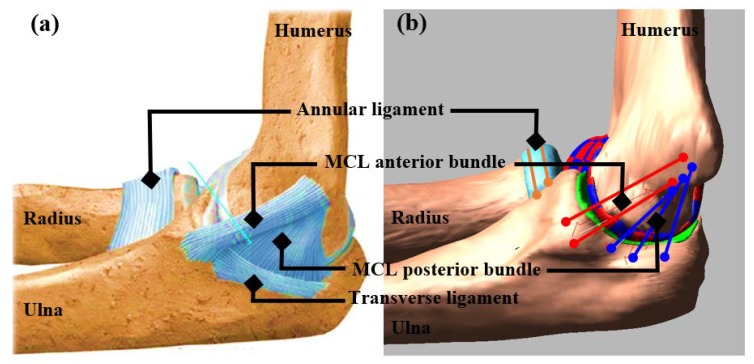
(**a**) Medial collateral ligament (MCL) complex consists of the MCL anterior bundle, MCL posterior bundle, and transverse ligament, (**b**) Corresponding ligament representation in the model.

Anatomically, the MCL complex consists of three components: The anterior, the posterior, and the transverse bundles (Figure 1a,b). The anterior bundle originates from the medial epicondyle of the humerus and attaches to the sublime tubercle on the coronoid process of the ulna. The posterior bundle also originates from the humeral medial epicondyle and generally inserts more posteriorly along the medial aspect of the olecranon. The transverse ligament originates from the ulna at the posterior side and inserts to the ulna anteriorly. This ligament currently has no known functions, and therefore, was not included in our study [19].

The primary objective of this study was to investigate medial collateral ligament deficiency of the elbow joint in computational multibody model simulation. Four cases were simulated in this study: (i) Keeping all ligament intact (‘Intact’ model); (ii) isolated MCL anterior bundle deficiency (MCL AB deficient); and (iii) isolated MCL posterior bundle deficiency (MCL PB deficient) (iv) and combined MCL anterior and posterior bundle deficiency (Both MCL deficient). For each ligament deficiency condition, kinematics, contact area, contact pressure, and ligament forces were calculated and compared to the intact model simulation.

## 2. Materials and Methods 

**Figure 2:**
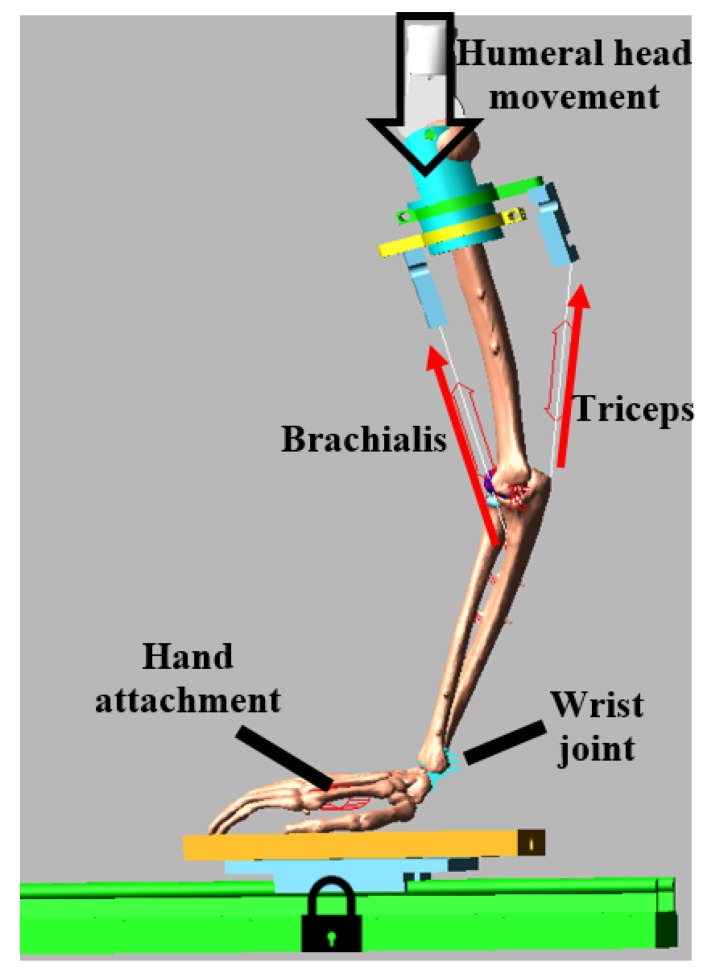
Computer model in ADAMS.

Two previously developed and evaluated multibody models created from two healthy cadaver specimens (Specimen #1, 61 years old, male, right arm; Specimen #2, 42 years old, male, right arm) were used for this study. The model development procedure is described in details in our previous publication [14]. Briefly, three-dimensional geometries of bones and cartilages were derived from computed tomography (slice thickness: 2 mm; Siemens Medical Solutions, PA, USA) and magnetic resonance images (Siemens 3T machine with a narrow field fine resolution setting; TR: 1200, TE: 38, slice thickness: 0.5 mm). The geometries were then imported to the commercial multibody dynamic software ADAMS (MSC Software Corporation, Santa Ana, CA) to generate the models (Figure 2). During experimental testing, multiple 3D points were recorded using Optotrack probe (Northern Digital Inc, Waterloo, ON, Canada) along the bone surface and bony landmarks at the desired initial arm position that later used as a reference to align our model geometries at the same initial position. The initial flexion angles were 20° for specimen #1 and 45° for specimen #2. Based on the total arm length and a certain machine height the initial flexion positions were different between specimens. It should not have a significant effect in our study since the investigation was done within the same range of motion. The forearm was placed in a full-pronated position. The model was constrained by attaching the humeral head with the ground through a transitional joint. The hand was placed on a metal plate and attached through a six-degree spring to the ground. Radius and hand were attached through a spherical joint to represent the wrist joint. Finally, a 345 mm vertical downward motion was applied on the humeral head for a total of 40 s to simulate elbow flexion. 

**Figure 3:**
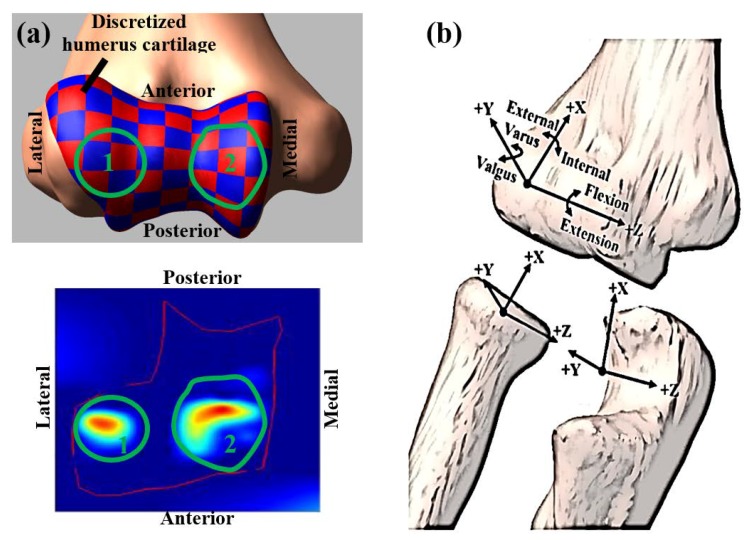
(**a**) Red and blue square represent the discretized pieces of humerus cartilage (top panel). The green circular region in top and bottom panel represents approximate contact region in 3D geometry and in the mapped 2D surface. (**b**) Joint coordinate system.

The humerus cartilage was discretized into multiple elements, and each discretized element had an approximate cross-sectional area of 5 × 5 mm (Figure 3a). This discretization provided an opportunity to calculate contact pressure distribution instead of single points contact. All elements were attached to the distal humerus bone surface with a fixed joint. The ulna and radius cartilages also were rigidly attached to the respective bone articulating surfaces using fixed joints. Deformable contact constraints were defined between each humerus cartilage element and the radius and ulna cartilage geometries using the modified Hertzian contact law (Equation (1)) [20].

(1) $$\;F_{c}=k_{c}\delta^{n}+B_{c}\left(\delta \right)\dot{\delta }\;$$

where *F_c_* is the contact forces, $k_{c}$ is the contact stiffness, *δ* is the interpenetration of the geometries, $\dot{\delta }$ is the velocity of interpenetration, *n* is the nonlinear power exponent, and $B_{c}\left(\delta \right)$ is a damping coefficient. The damping coefficient is defined as a function of interpenetration to prevent discontinuities in the forces of the first contact and defined as [21]:

(2) $$\;B_{c}\left(\delta \right)=\{\begin{array}{cc} 0 & \delta \leq 0\\ B_{max}{\left(\frac{\delta }{d_{max}-\delta }\right)}^{2}\left(3-\frac{2\delta }{d_{max}-\delta }\right) & 0<\delta <d_{max}\\ B_{max} & \delta >d_{max} \end{array}\;$$

where $d_{max}$ is the penetration at which the maximum damping $B_{max}$ is applied.

Elastic foundation theory was used to estimate the contact parameters [6,22].

(3) $$p=\frac{\left(1-\nu \right)E}{\left(1+\nu \right)\left(1-2\nu \right)h}d\;$$

here *E* is Young’s modulus, ν is Poisson’s ratio, *h* is the combined cartilage thickness in articulation, and *d* is the spring deformation. The contact pressure *p* was computed for the values of *E* = 0.7 MPa [23], *ν* = 0.495 [16], *h* = 4, 4.8, 3.07, 3.58 [14] (for humerus–ulna and humerus–radius articulation of specimen 1 and 2 respectively) with *d* as an unknown spring deformation. Since the humerus cartilage was discretized in 5 × 5 mm, the value of *p*/*d* was then multiplied by 25 mm^2^ to estimate contact stiffness. The final values used in Equations (1) and (2) are: $k_{c}$ = 126 N/mm (ulnohumeral) & 105 N/mm (radiohumeral), N = 1, $B_{max}$ = 2 Ns/mm, and $d_{max}$ = 0.1 [13]. Based on our previous experience, these values of n, $B_{max}$, and $d_{max}$ work well for the dynamic simulation without any significant alteration effect on joint kinematics and contact characteristics. 

The ligaments and interosseous membranes were represented as single force elements with nonlinear force-strain curves including the non-linear “toe” region. The model included three bundles each for the MCL anterior and posterior band, three bundles for the lateral ulnar collateral ligament (LUCL), three bundles for the radial collateral ligament, and two bundles for the annular ligament. The force-length relationship of each ligament is described by [22]:

(4) $$f=\{\begin{array}{cc} \frac{1}{4}k\varepsilon^{2}/\varepsilon_{l} & 0\leq \varepsilon \leq 2\varepsilon_{l}\\ k\left(\varepsilon -\varepsilon_{l}\right) & \varepsilon >2\varepsilon_{l}\\ 0 & \varepsilon <0 \end{array}\;$$

(5) $$\varepsilon =\left(\frac{l-l_{0}}{l_{0}}\right)\;$$

where *k* is a stiffness parameter, *ɛ* is the engineering strain of each ligament part, $\varepsilon_{l}$ is a spring parameter assumed to be 0.03 [24], *l* is the ligament bundle length, and *l*_0_ is the zero-load length. The stiffness parameters *k* was obtained from Fisk et al. [7] and Regan et al. [25]. The *l*_0_ was estimated based on previous cadaveric studies performed by Rahman et al. [14,26]. Each ligament force also had a parallel damper with a damping coefficient of 0.5 Ns/mm to remove the possibility of high-frequency vibration during simulation [10]. LUCL and annular ligaments were wrapped around the bone for better representing the ligament force’s lines of action [14]. Constant muscle tension of 40 N for the triceps and 20 N for the brachialis were applied in the model throughout the simulation period to provide some muscle stabilization [7,27]. Bicep muscle tension was not simulated since the experimental study had not included it in the design. 

Simulated bone motions were measured by defining local coordinate systems for each bone segment as described by Ferreira et al. [28] and Morrey et al. [29] (Figure 3b). The three translations and rotations of the radius and ulna were computed relative to the humerus. The translations were calculated at medial-lateral (M-L), anterior-posterior (A-P), and superior-inferior (S-I) direction and the rotations were calculated at flexion-extension (F-E), varus-valgus (VR-VL), and internal-external (I-E) rotation. The vertical downward motion was applied to the humeral head to induce a maximum flexion angle of about 135° for both specimens. All ligament deficient conditions and the intact elbow were subjected to the same motion profile for a particular specimen. 

One-way analysis of variance (ANOVA) was performed with ligament state as a variable to observe the kinematic difference between the intact and different ligament deficient case. Then, Tukey-Kramer multiple pairwise comparison test was used to find the significant difference between the intact and other ligament condition. Significance was defined at *p* ≤ 0.01. Kinematic differences were calculated for every two degrees of flexion angle from the range of 50° to 130° flexion to get data points. Thus, within this 80° flexion range, a total of 40 sample data points was obtained for each ligament condition to perform the statistical analysis. 

## 3. Results

### 3.1. Kinematic Comparison

**Figure 4:**
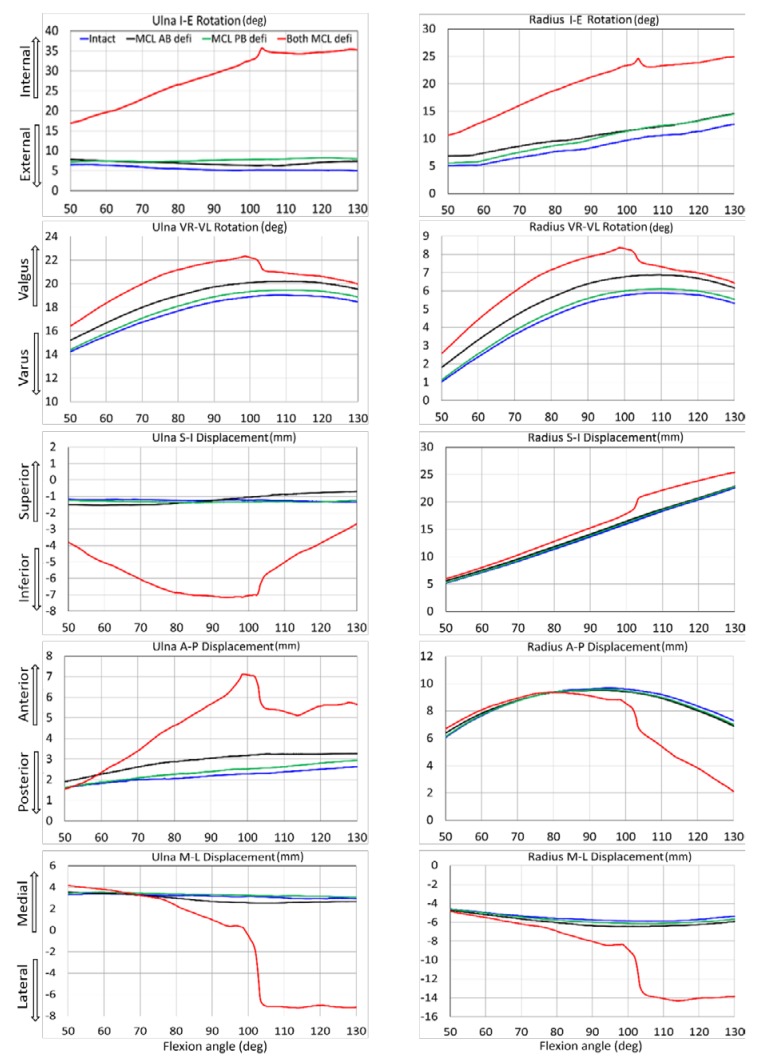
Effect of medial collateral ligament deficiency on ulna and radius kinematics relative to humerus for specimen 1.

**Figure 5:**
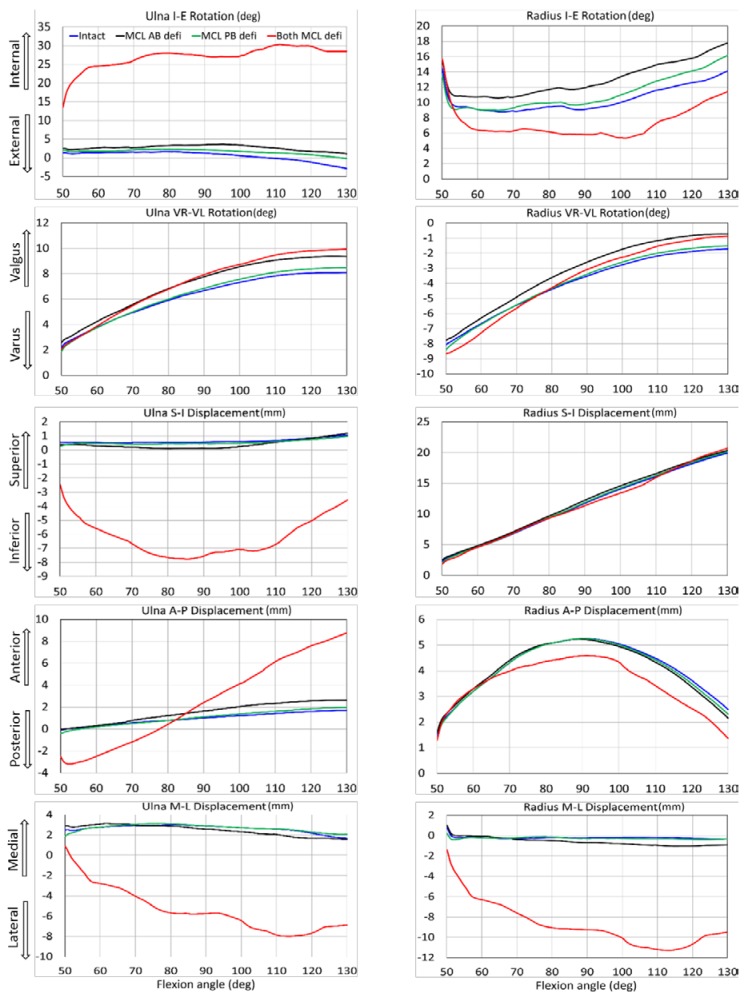
Effect of medial collateral ligament deficiency on ulna and radius kinematics relative to humerus for specimen 2.

For both specimens, several ulna and radius kinematics were significantly different for isolated MCL AB and PB deficiency (Table 1 and Table 2). Sectioning both bundles together generated significant differences in almost all elbow joint kinematics. Kinematic observations for different ligament conditions are presented for both specimens from 50° to 130° flexion range (Figure 4 and Figure 5). When the entire MCL bundle was sectioned, maximum ulna internal rotation was more than 30° at deep flexion, indicating significant elbow instability. With that, the ulna was also laterally translated for more than 8 mm.

**Figure 6:**
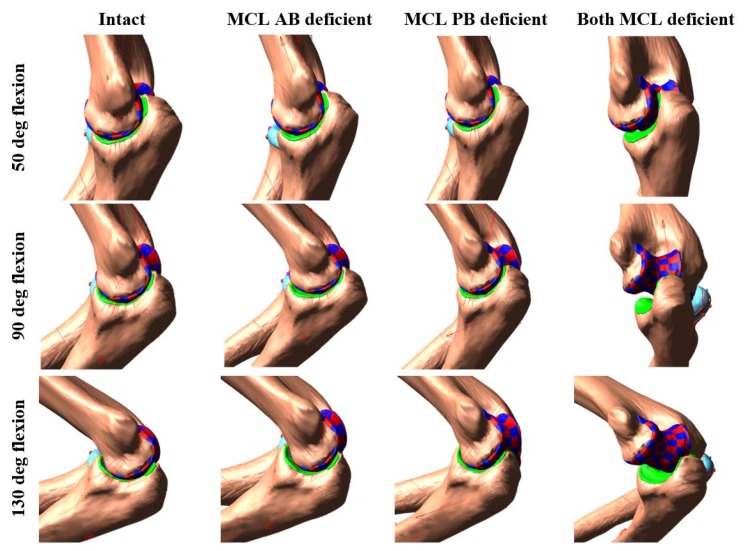
Elbow joint configuration at different flexion angles for specimen 1.

**Figure 7:**
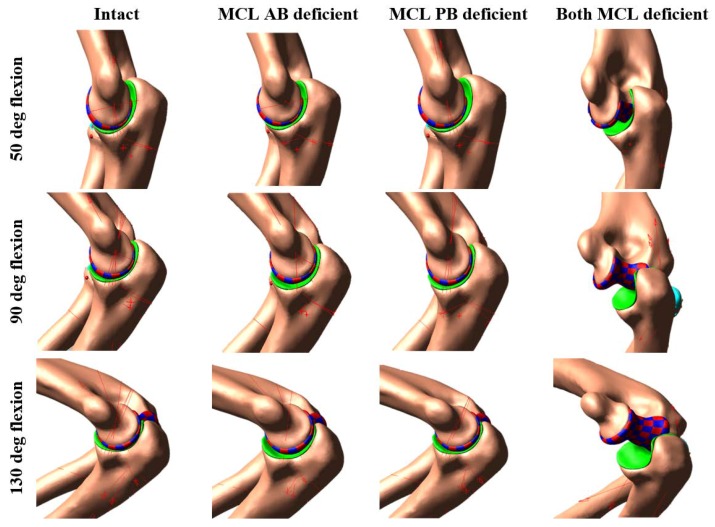
Elbow joint configuration at different flexion angles for specimen 2.

Complete elbow dislocations were not observed for any ligament deficient cases. However, the joint was significantly distracted at around 80° of flexion when the entire MCL bands were sectioned and continuously reduced by increasing the flexion angle (Figure 6 and Figure 7).

### 3.2. Contact Area and Pressure Comparison

**Figure 8:**
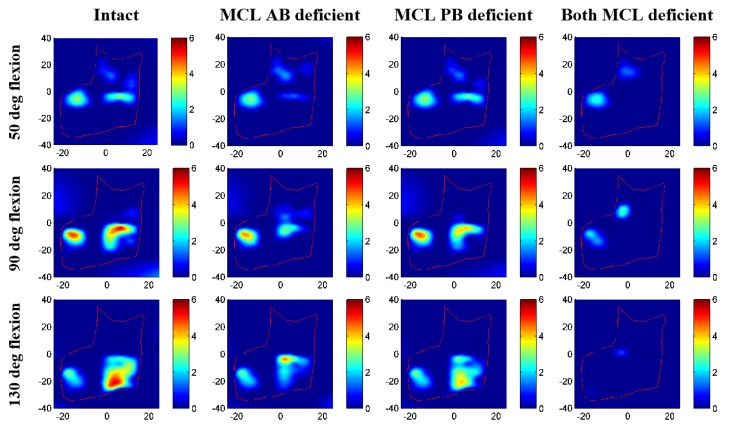
Contact pressure distribution on humerus cartilage for specimen 1.

**Figure 9:**
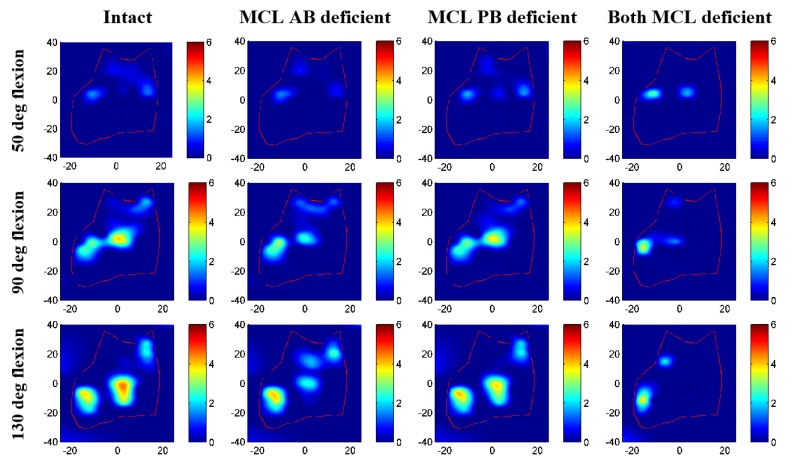
Contact pressure distribution on humerus cartilage for specimen 2.

Contact pressure distributions on humeral cartilage changed noticeably for different ligament deficient conditions compared to intact elbow (Figure 8 and Figure 9). Contact patches were almost identical for intact and MCL PB deficient elbow; minor differences were observed for the MCL AB deficient case. The most significant change in contact characteristics occurred for the entire MCL deficient case for both specimens. In this case, the joint contact was significantly absent on the medial cartilage indicating significant distraction of the joint. Maximum contact pressure for specimen 1 was 5.2 MPa for intact, 4.4 MPa for MCL AB, 4.3 MPa for MCL PB, and 2.8 MPa for both MCL deficient cases. For specimen 2, maximum contact pressure was 4.4 MPa for intact, 3.9 MPa for MCL AB, 3.7 MPa for MCL PB, and 3.7 MPa for both MCL deficient case. Maximum contact patches were seen for intact cases and almost disappeared for both MCL deficient conditions. 

### 3.3. Ligament Load Comparison

Peak loads for the MCL PB ligament considerably increased for the MCL AB deficient condition (Table 3 and Table 4). Similarly, for the MCL PB deficient elbow, peak loads for MCL AB ligament increased. Peak loads for other intact ligaments substantially decreased for complete MCL deficiency. Flexion angle, in which the maximum ligament load occurs, was highly variable but mostly at high flexion angle (more than 110°).

## 4. Discussion

This study investigated the elbow joint behavior during passive flexion in the presence of medial collateral ligament deficiency in a computational model. Two previously developed anatomically based computational multibody elbow joint models were used for this study [14]. The computational models were created from the subject-specific bones and cartilage geometries derived from medical images and evaluated through experimental cadaver tests. The joints were constrained by non-linear ligaments including ligament zero load length. The ligaments were wrapped around the joint for better representation of ligament physiology and line of action. The model includes discretized humeral cartilage that allows computation of contact pressure distribution and contact area. 

Several studies have examined the effect of a medial collateral ligament injury in the human elbow joint by using in vitro cadaver experiments [1,4,30]. However, computational model investigations of these injuries are very limited in the literature. The advantages of computer models are that they provide flexibility in analyzing different clinical scenarios and can predict what cannot be directly measured, such as ligament loading and cartilage contact pressure. Such information would allow the pre-operative assessment of which ligament should actually be reconstructed, and the anticipated cartilage loading to prevent cartilage degeneration and osteoarthritis. Better pre-operative planning would allow for the most effective treatment and possibly less invasive surgery of the elbow injury and may lead to the development of newer elbow ligament reconstruction techniques.

On average, in our study, the mean kinematic differences for MCL AB deficient elbow were slightly larger than MCL PB deficient conditions (Table 1 and Table 2). This observation indicates that MCL AB ligament has a somewhat bigger influence on kinematic constraints than MCL PB ligament. However, the joint was entirely in contact over the simulation period for both cases, as there was no valgus stress applied to the elbows. The joint was significantly distracted only when the entire MCL bundle was sectioned even if there was no valgus stress. The kinematics was slightly varied between the two specimens. Besides, the ulna translated more laterally for specimen #1 at about 100° flexion, causing a sudden spike in results. The reason for this variation could be described as some geometrical variation between the subject. Specimen 2 had smooth, better shaped, and a broader joint surface than specimen #1, and thereby provided some better accuracy for joint articulation and choosing boney landmark. Due to the variability between the subjects, other results were also slightly varied.

Our study observed that when the entire MCL bundle was disrupted, ulna generated the maximum internal rotation of about 30–35° (Figure 4 and Figure 5). This result was in good agreement with the cadaveric experimental study reported by Armstrong et al. [4]. In their study, they demonstrated that for a passive motion of a pronated forearm, the arm rotated internally about 30–35° when the entire MCL bundle was sectioned. However, their reported maximum varus-valgus laxity was about 40–50°, which was higher than our observed maximum valgus rotation of about 20°. The reason for this discrepancy was the difference between their testing system and our model boundary conditions. In their experimental setup, the arm was placed in a gravity loaded position to observe the varus-valgus laxity after sectioning MCL. On the other hand, we placed the arm vertically and simulated elbow flexion by providing a vertical motion to the humeral head. 

None of the ligament deficient conditions induced complete elbow dislocation (Figure 6 and Figure 7). However, entire MCL bundle deficiency significantly reduced elbow stability. Ligament deficiency also induced significant changes to the contact pattern over the simulation period (Figure 8 and Figure 9). Contact area patterns were almost identical for the intact and the MCL PB deficient condition over the simulation period, but this ligament deficiency induced reduction of the peak joint contact pressure, signifying a small amount of joint laxity. On the other hand, contact area pattern and peak contact pressure were markedly different for the MCL AB deficient elbow compared to the intact elbow, indicating joint subluxation. The most critical change in the contact area occurred in both deficient cases with the significant loss of contact in the medial humerus and medial joint distraction. However, the radial head kept contact with the capitellum for all cases.

Peak ligament loads increased for the MCL posterior bundle when the MCL anterior bundle was sectioned (Table 3 and Table 4). This result indicates that in the absence of the anterior bundle, the posterior bundle carried part of the joint load from the anterior side. A similar trend was observed for the MCL PB deficient case where the anterior bundles loads were increased in the absence of the posterior bundle. Peak ligament loads of the lateral collateral ligament complex were less influenced by the MCL deficiency. When the entire MCL bundle was removed peak load decreased in almost all intact ligaments. This is because, in this ligament deficiency condition, the elbow joint opens up on the medial side and causes a slack on the lateral side.

A limitation of the present study was that the model did not include any joint capsule and all muscles crossing the elbow joint. Thus, the stabilizing influences from these tissues were not quantified in this study. An et al. [31] and King et al. [32] reported that muscle loading applied to the elbow during active motion allows axial compressive forces at the joint to enhance joint stabilization. Our study analyzed passive flexion, so muscle stabilizing effects may be less influential. Morrey et al. [33] reported that at full extension, the anterior and posterior joint capsule provided 32% varus and 33% valgus elbow stability, respectively. De Hann et al. [34] reported that the joint capsule severs as a secondary static elbow stabilizer when the elbow is extended. Our model did not include the joint capsule contribution to joint stability, and therefore laxity effects could be exaggerated. Mechanical properties of elbow joint capsules are not available in the current literature which deterred us from incorporating it into our model. Future studies will incorporate elbow-crossing muscles along with the capsule’s stability in the joint. 

Secondly, the discretized cartilage contact parameters and discrete cartilage size were not optimized for the current model and estimated based on simplified elastic foundation theory. Willing et al. [16] reported that accurate selection of the material properties is significant for calculating cartilage contact pressures when using a finite element model. Our models were based on rigid body dynamics. However, future studies will also optimize contact parameters and discretized cartilage size by matching a multibody cartilage model with a finite element model of the same cartilage [35,36]. Even with this limitation, our maximum contact pressures were close in range (0.5–5 Mpa) to the values reported in the literature [17,37]. The predicted intact contact areas at 20° flexion were in good agreement with the results reported by Willing et al. [16]. Furthermore, our predicted contact and non-contact areas of ulnohumeral articulation were consistent with the contact patterns reported by Eckstein et al. [38]. Lastly, this study included two specimens and characterized two subjects. Larger sample size could be helpful for more generalized conclusion and statistical significant. However, our research team has extensive expertise in developing subject-specific model form the both cadaver and live subject. 

The primary objective of this study was to simulate different combinations of MCL complex deficiency in a computer model. For each simulation, we measured and compared the joint kinematics, contact characteristics (area and pressure), and intact ligament load. Our results indicate that either MCL AB or MCL PB can provide anterior stability of the elbow joint during passive flexion. However, the MCL AB has a somewhat bigger influence on joint kinematics and contact characteristics compared to the MCL PB. Sectioning both bundles together induced significant joint disruption. For the complete MCL injuries, reconstructing only the anterior bundle of the MCL is consider as a successful clinical practice [39,40]. Our results indicate that intact isolated MCL anterior bundle may not fully retrieve its normal joint contact characteristic which could be an influencing factor of postoperative osteoarthritis development in the elbow joint. A further study with larger sample size is required to strengthen the conclusion. Detailed knowledge of the effects of the MCL complex deficiency can be of great importance to Orthopaedic surgeons planning surgical approaches to the medial aspect of the elbow for ligament or fracture repairs. It could also be immensely useful for post-operative rehabilitation protocols. 

## Figures and Tables

**Table 1:** Mean kinematics difference over the range of flexion ± standard deviation (statistical *p*-values) between ligament deficient and intact elbow for specimen 1.

	Ligament Conditions	I-E (deg)	VR-VL (deg)	S-I (mm)	A-P (mm)	M-L (mm)
Ulna Kinematics	MCL AB Deficient	1.44 ± 0.36	1.18 ± 0.08	0.10 ± 0.35	0.73 ± 0.17	−0.28 ± 0.23
		(0.19)	(<0.01 *)	(0.93)	(<0.01 *)	(0.94)
	MCL PB Deficient	2.24 ± 0.73	0.40 ± 0.08	−0.08 ± 0.06	0.20 ± 0.09	0.15 ± 0.04
		(0.02)	(<0.01 *)	(0.96)	(0.49)	(0.99)
	Both MCL Deficient	23.72 ± 6.50	2.61 ± 0.76	−4.19 ± 1.46	2.61 ± 1.26	−4.71 ± 4.62
		(<0.01 *)	(<0.01 *)	(<0.01 *)	(<0.01 *)	(< 0.01 *)
Radius Kinematics	MCL AB Deficient	1.91 ± 0.16	0.97 ± 0.07	0.43 ± 0.07	−0.13 ± 0.19	−0.47 ± 0.15
		(<0.01 *)	(<0.01 *)	(<0.01 *)	(0.95)	(0.61)
	MCL PB Deficient	1.41 ± 0.47	0.22 ± 0.03	0.20 ± 0.05	−0.11 ± 0.11	−0.21 ± 0.90
		(<0.01 *)	(<0.01 *)	(0.41)	(0.97)	(0.94)
	Both MCL Deficient	11.42 ± 2.25	1.93 ± 0.57	2.24 ± 1.14	−1.78 ± 2.13	−4.21 ± 3.44
		(<0.01 *)	(<0.01 *)	(<0.01 *)	(<0.01 *)	(<0.01 *)

I-E = internal-external rotation; VR-VL = varus-valgus rotation; S-I = superior-inferior translation; A-P = anterior-posterior translation; M-L = medial-lateral translation. Positive values indicate more internal for I-E, and more valgus rotation for VR-VL than the intact elbow. Similarly, positive values indicate more superior for S-I, more anterior for A-P, and more medial translation for M-L than the intact elbow. Negative values indicate the opposite. The asterisk (*) for *p*-values indicates significance (*p* ≤ 0.01).

**Table 2:** Mean kinematics difference over the range of flexion ± standard deviation (statistical *p*-values) between ligament deficient and intact elbow for specimen 2.

	Ligament Conditions	I-E (deg)	VR-VL (deg)	S-I (mm)	A-P (mm)	M-L (mm)
Ulna Kinematics	MCL AB Deficient	2.29 ± 0.79	0.99 ± 0.32	−0.23 ± 0.17	0.61 ± 0.35	−0.21 ± 0.31
		(<0.01 *)	(<0.01 *)	(0.38)	(0.35)	(0.78)
	MCL PB Deficient	1.17 ± 0.64	0.17 ± 0.16	−0.10 ± 0.03	0.09 ± 0.14	0.05 ± 0.14
		(0.03)	(0.13)	(0.89)	(0.99)	(0.99)
	Both MCL Deficient	26.89 ± 3.78	4.14 ± 0.62	−6.87 ± 1.27	1.97 ± 3.33	−8.31 ± 2.01
		(<0.01 *)	(<0.01 *)	(<0.01 *)	(<0.01 *)	(<0.01 *)
Radius Kinematics	MCL AB Deficient	2.73 ± 0.77	0.82 ± 0.26	0.41 ± 0.10	−0.07 ± 0.14	−0.43 ± 0.34
		(<0.01 *)	(<0.01 *)	(<0.01 *)	(0.47)	(0.24)
	MCL PB Deficient	0.84 ± 0.66	0.10 ± 0.12	0.12 ± 0.10	−0.05 ± 0.06	−0.07 ± 0.11
		(<0.01 *)	(0.06)	(0.41)	(0.73)	(0.99)
	Both MCL Deficient	−3.22 ± 1.17	0.28 ± 0.49	−0.05 ± 42	−0.68 ± 0.41	−8.83 ± 2.06
		(<0.01 *)	(<0.01 *)	(0.70)	(<0.01 *)	(<0.01 *)

I-E = internal-external rotation; VR-VL = varus-valgus rotation; S-I = superior-inferior translation; A-P = anterior-posterior translation; M-L = medial-lateral translation. Positive values indicate more internal for I-E, and more valgus rotation for VR-VL than the intact elbow. Similarly, positive values indicate more superior for S-I, more anterior for A-P, and more medial translation for M-L than the intact elbow. Negative values indicate the opposite. The asterisk (*) for *p*-values indicates significance (*p* ≤ 0.01).

**Table 3:** Maximum intact ligament loads and strains for specimen 1.

Ligament Band	Intact		MCL AB Deficient		MCL PB Deficient		Both MCL Deficient	
	Peak Load (N)	Max. Strain	Peak Load (N)	Max. Strain	Peak Load (N)	Max. Strain	Peak Load (N)	Max. Strain
Lateral ulnar collateral ligament	62	0.13	59	0.13	54	0.12	44	0.10
Radial collateral ligament	24	0.11	20	0.10	19	0.10	23	0.11
MCL anterior band	211	0.50	-	-	224	0.53	-	-
MCL posterior Band	118	0.44	140	0.52	-	-	-	-

The missing values mean the ligaments are sectioned and providing no constraints.

**Table 4:** Maximum intact ligament loads and strains for specimen 2.

Ligament Band	Intact		MCL AB Deficient		MCL PB Deficient		Both MCL Deficient	
	Peak Load (N)	Max. Strain	Peak Load (N)	Max. Strain	Peak Load (N)	Max. Strain	Peak Load (N)	Max. Strain
Lateral ulnar collateral ligament	53	0.11	45	0.10	49	0.11	31	0.08
Radial collateral ligament	20	0.10	16	0.09	18	0.09	43	0.18
MCL anterior band	170	0.40	-	-	181	0.43	-	-
MCL posterior band	65	0.26	95	0.36	-	-	-	-

The missing values mean the ligaments are sectioned and providing no constraints.
